# Supplementary material for: High-Throughput Identification of MiR-145 Targets in Human Articular Chondrocytes
Source: Life (Basel). 2020 May 11;10(5):58. doi: 10.3390/life10050058 (PMC7281014; doi:10.3390/life10050058)
Supplement: Supplementary file 1 [file life-10-00058-s001.zip › life-774119 Supplemental for XML/life-774119 Supp Figures.pdf]

A

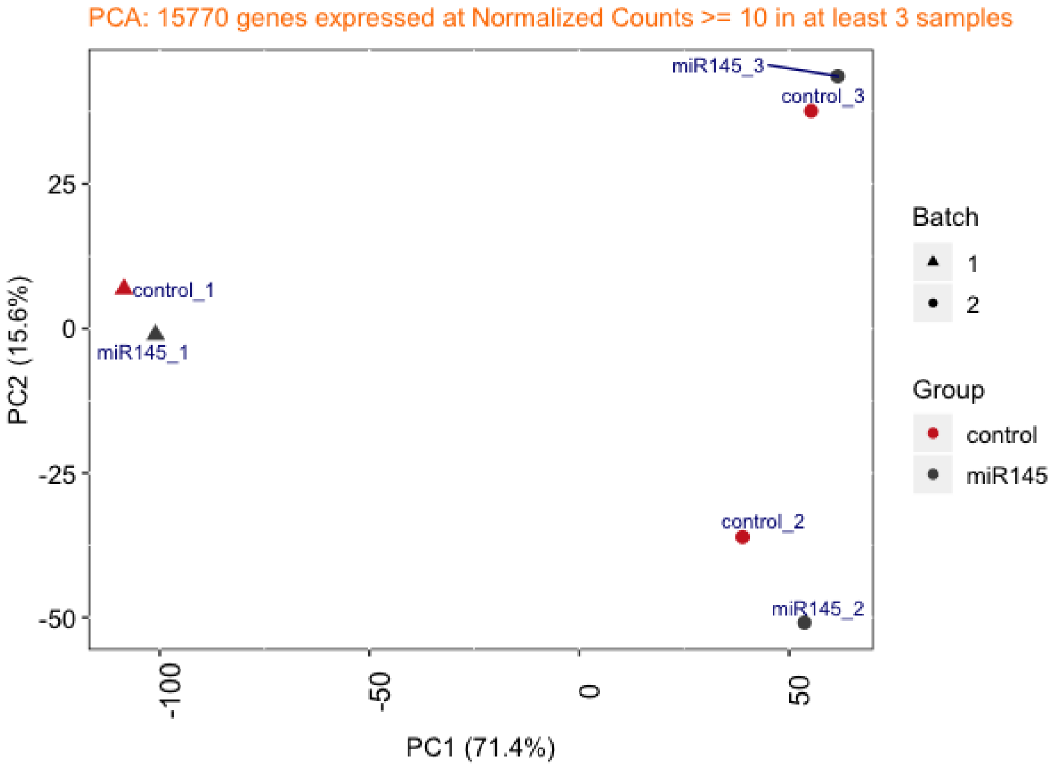

B

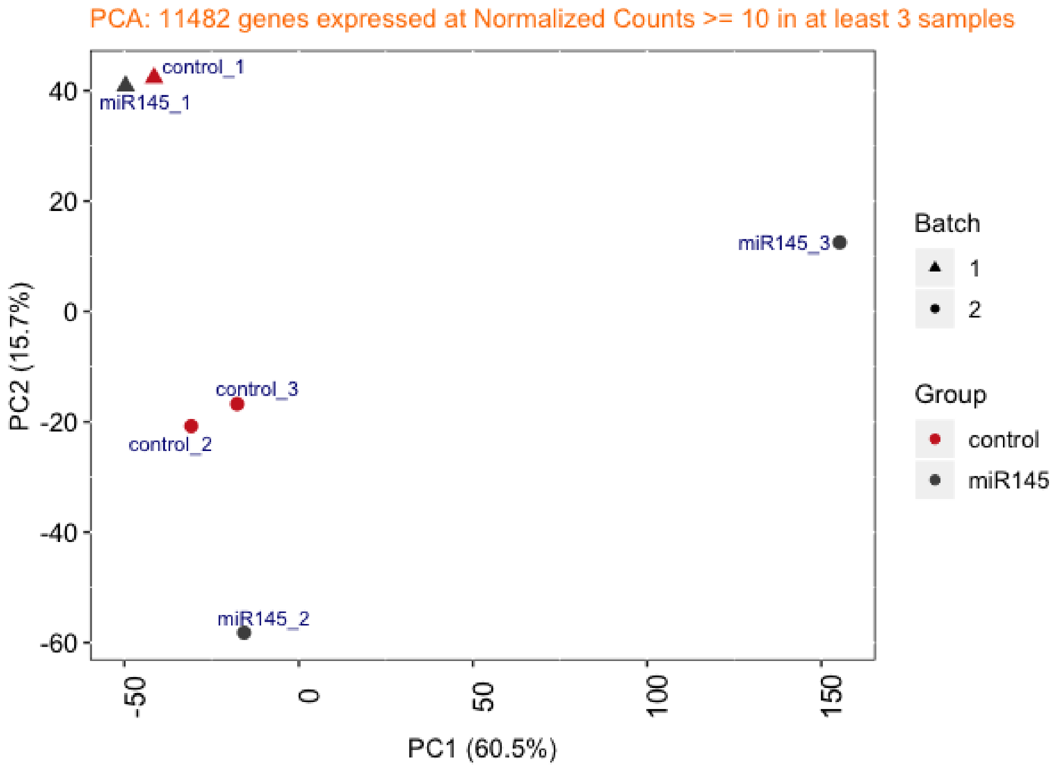

Supp Figure 1. Principal component analysis (PCA) of the samples used for total mRNA seq (A, T-RNA) and RIP-seq (B, IP-RNA) in this study. The PCA shows the batch effect, sample pairing and treatment. Samples are numbered by HACs donor (1-3, independent HACs donors) and color-coded by treatment (control (red) and miR-145 (black) mimics. Samples were obtained in two separate experiments, indicated by batch (triangle: experiment 1, circle, experiment 2). Note that the highest variation is explained by the HACS donor for total RNA but not for IP-RNA. One sample in the IP-RNA group (miR-145_3, “Outlier”) separates from the rest of the data.

A
IP-RNA (CDS)

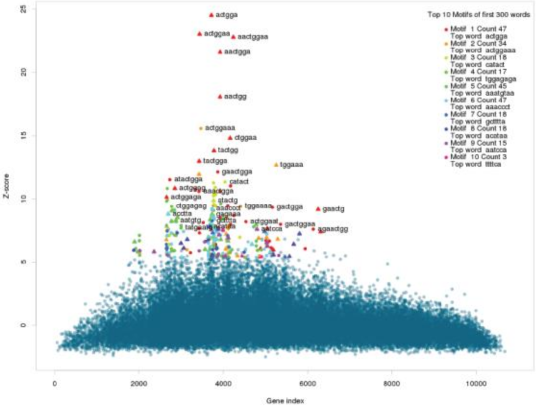

B
IP-RNA (CDS)

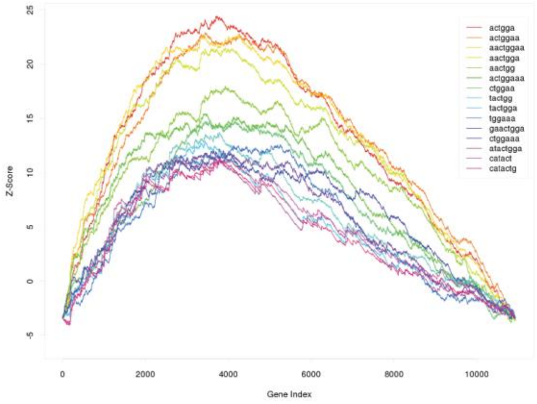

C
IP-RNA (3'UTR)

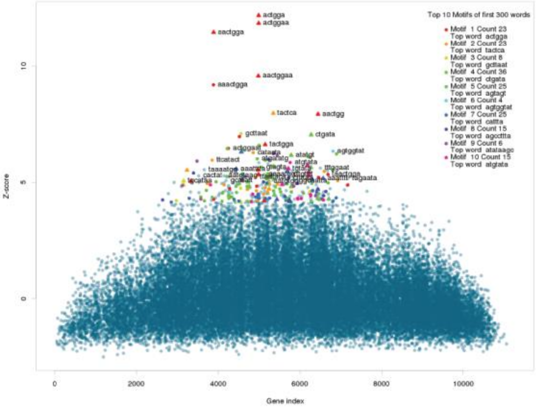

D
IP-RNA (3'UTR)

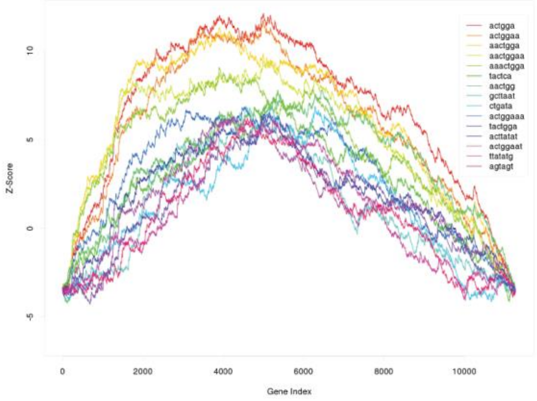

Supp Figure 2. . Enrichment of miR-145 seed sites in the CDSs (A, B) or 3'UTRs (C, D) of the precipitated mRNAs (outlier excluded, only 2 experiments). A, C) Scatter plot showing maxima of enrichment profile. Each dot represents a word, the Y-position reflects the maximum score of an enriched word and X-position shows the gene-index where the Z-score is maximum. Top-ranked words with left-shift associate with gene-expression change (lower gene index represent stronger up-regulation in Ago2 upon miR-145 transfection). Triangles annotate known seed sites of human miRNAs, red triangles show miR-145 seed sites. **B, D)** Word-enrichment profile: the line plot shows enrichment through the gene rank for the top ten enriched words. Each line represents the running sum over all scores that quantify degree of enrichment according to gene up-regulation (from most up-regulated to most-downregulated)
